# Raw Beef Patty Analysis Using Near-Infrared Hyperspectral Imaging: Identification of Four Patty Categories

**DOI:** 10.3390/s23020697

**Published:** 2023-01-07

**Authors:** Kiah Edwards, Louwrens C. Hoffman, Marena Manley, Paul J. Williams

**Affiliations:** 1 Department of Food Science, Stellenbosch University, Private Bag X1, Matieland, Stellenbosch 7602, South Africa; 2 Department of Animal Sciences, Stellenbosch University, Private Bag X1, Matieland, Stellenbosch 7602, South Africa; 3 Centre for Nutrition and Food Sciences, Queensland Alliance for Agriculture and Food Innovation (QAAFI), The University of Queensland, Health and Food Sciences Precinct, 39 Kessels Rd, Coopers Plains 4108, Australia

**Keywords:** meat fraud, processed meat, near-infrared hyperspectral imaging, chemometrics, machine learning

## Abstract

South African legislation regulates the classification/labelling and compositional specifications of raw beef patties, to combat processed meat fraud and to protect the consumer. A near-infrared hyperspectral imaging (NIR-HSI) system was investigated as an alternative authentication technique to the current destructive, time-consuming, labour-intensive and expensive methods. Eight hundred beef patties (*ca.* 100 g) were made and analysed to assess the potential of NIR-HSI to distinguish between the four patty categories (200 patties per category): premium ‘ground patty’; regular ‘burger patty’; ‘value-burger/patty’ and the ‘econo-burger’/’budget’. Hyperspectral images were acquired with a HySpex SWIR-384 (short-wave infrared) imaging system using the Breeze^®^ acquisition software, in the wavelength range of 952–2517 nm, after which the data was analysed using image analysis, multivariate techniques and machine learning algorithms. It was possible to distinguish between the four patty categories with accuracies ≥97%, indicating that NIR-HSI offers an accurate and reliable solution for the rapid identification and authentication of processed beef patties. Furthermore, this study has the potential of providing an alternative to the current authentication methods, thus contributing to the authenticity and fair-trade of processed meat products locally and internationally.

## 1. Introduction

Adulteration of food has a long history and is still a common practice around the world. Food adulteration occurs to increase financial return, where a high-value food is contaminated by adding a low-value ingredient. Adulteration is not only a form of consumer fraud and economic misconduct, but it also poses a risk to people who are allergic to certain ingredients or meat species, have religious prohibitions, or have ethical aversions [1]. Furthermore, it seriously violates the rights of consumers.

Meat adulteration is a current issue with economic and safety implications because it is difficult to detect specific ingredients/adulterants and/or distinguish between species when evaluating meat visually [2]. In the past, meat adulteration scandals have included undeclared soy protein incorporation in Brazilian hamburgers [3], meat from undeclared animal species in Mexican hamburgers and sausages [4], undeclared animal species in various meat products in the USA [5], and Turkey [6], and the incorporation of undeclared horsemeat in processed beef products in the EU [7,8]. In South Africa, Cawthorn et al. [9] discovered species such as chicken, goat, water buffalo, and donkey in beef sausages that were not disclosed on the product labelling. Following such reports, consumers became concerned about the traceability and origin of the food they eat.

Therefore, in an attempt to protect the consumer, the South African government proposed new regulations [10] stipulating the composition and what constitutes a ‘beef patty’. These specify that patties are divided into four categories: premium ‘ground patty’ (99.6% meat); regular ‘burger patty’ (70% + meat); ‘value-burger/patty’ (55% meat with ≤30% fat) and the ‘econo-burger’/’budget’ (35% meat with ≤30% fat). The ‘value-burger’ and ‘econo-burger’ may also contain vegetable protein, mechanically recovered meat and edible offal. Thus, it is critical that these meat products are labelled correctly and reliably so that consumers may make educated decisions when purchasing these products. These new regulations aim to combat processed meat fraud. However, the current conventional techniques require a human inspector to sample between 250 g and 2 kg of a primary sample, depending on the capacity of the container (Table S1), prepare the test samples in accordance with the Official AOAC Method 983.18 and then determine the composition of the raw processed meat products according to the methods set out in Table S2 [10]. These techniques are destructive, time-consuming, labour-intensive and can easily overwhelm the human inspector performing the various analyses. Therefore, the non-destructive automation of this process is a feasible solution to avoid potential errors associated with the lack of human concentration and fatigue, and will allow for the rapid, continuous analysis of samples resulting in a higher throughput and larger bulk primary samples. Although conventional NIR spectroscopy could be used for similar applications, it only provides spectral information of a limited area of a sample, whereas spectral imaging provides spatial and spectral information of the entire scanned sample. Therefore, to cover a larger area of the sample with conventional NIR spectroscopy, multiple measurements are required which in turn will result in an increase in measurement time. Collecting spectral information from the entire sample would thus be difficult.

Hyperspectral imaging has become one of the most effective and advanced non-destructive evaluation technologies for a wide range of applications. Hyperspectral imaging combines imaging and spectroscopy in a single instrument to obtain both spatial and spectral data from an object at the same time [11,12]. This technique is gaining popularity due to its advantages over other instrumental techniques. Due to rising quality and safety concerns, the capacity to detect individual molecules, compounds, or substances within samples has become critical. NIR-HSI can instantly (at-line) and continuously (on-line) record spectra of samples to predict chemical and physical properties from a single measurement [13,14]. These characteristics enable quick, non-destructive sample examination, potentially allowing for improved quality control and process monitoring in a factory setting, particularly for on-line and at-line applications over a conveyer belt.

A number of researchers have investigated the use of HSI for meat and processed meat products authentication and studies have successfully been conducted to grade and classify pork [15], to discriminate between three lamb muscles [16], the discrimination between pork, beef and lamb meat [17,18], and the discrimination between fresh and frozen-thawed pork [19,20], the detection and quantification of pork adulteration in minced lamb [2], to detect and quantify the level of horsemeat adulteration in minced beef [21], the detection of duck meat adulteration in minced lamb [22], and to predict the adulteration level of minced pork with pork jowl meat [23].

From these studies it is evident that a plethora of research has been done on the authentication of meat and meat products with regard to the detection and quantification of various species adulterants. Despite the broad availability of literature on NIR-HSI applied to meat, no studies were found for classifying patties into different categories based on specific ingredients, species and adulterants [textured vegetable protein and/or mechanically recovered meat (MRM)]. Given the advances in NIR-HSI, chemometrics and machine learning, we hypothesise that this technique will be able to distinguish between the four patty categories. The information obtained could provide the South African and global meat sector with an alternative to the existing laborious, destructive, and time-consuming methods of detecting fraud, hence promoting the authenticity and fair trading of processed meat products both locally and internationally.

The aim of this study was to establish if NIR-HSI combined with image analysis, multivariate techniques and machine learning algorithms was capable of rapidly and accurately distinguishing between the four processed beef burger patty categories, consisting of various ingredients and adulterants (pork, lamb, ostrich, textured vegetable protein and MRM) at selected levels, irrespective of the treatments or the authenticity status.

## 2. Materials and Methods

### 2.1. Experimental Design

The study consisted of four formulations (Table 1, Table 2, Table 3 and Table 4), based on the four patty categories, of 200 patties each and for each formulation there was ten or five treatments (Figures S1–S4). The formulations and treatments were developed according to the South African regulations regarding the classification and compositional specifications of raw beef patties [10], shown in Table S3.

### 2.2. Beef Patty Production

#### 2.2.1. Ingredient Sourcing

The lean meat [beef (*Bos taurus*), pork (*Sus scrofa domesticus*), lamb (*Ovis aries*), ostrich (*Struthio camelus*)] and fat samples were obtained from Birdstreet Butchery (Stellenbosch, South Africa), in October 2020, vacuum sealed and stored at 4 °C until used. The meat consisted of deboned and defatted muscles while the fat comprised of subcutaneous fat trimmed from beef carcasses. The lean meat and beef fat were derived from numerous animal carcasses. The mechanically recovered meat (MRM) [poultry (*Gallus gallus domesticus*)] was obtained from Deli Species (Cape Town, South Africa), vacuum sealed and frozen at −20 °C until needed. The Burger Ready spice packs, burger rusk, textured vegetable protein (TVP) and soya fibre were also supplied by Deli Spices.

#### 2.2.2. Production

A total of 800 patties were produced at Deli Spices according to the different formulations and treatments shown in Table 1, Table 2, Table 3 and Table 4. Prior to patty production, the beef, pork, lamb, ostrich, MRM and fat were separately minced with a meat mincer (Mainca PC-32, Equipamientos Cárnicos, Barcelona, Spain) using 4.5 mm plate openings. All the ingredients for each patty formulation and treatment was thoroughly mixed in separate mixing bowls to ensure the even distribution of meat, fat and other added ingredients in the patty batter. A Butcherquip hand operated patty machine (Butcherquip, Roodepoort, South Africa) was used to press the patty batter into 100 g patties (21 mm thick) (Deli Spices), which were then packaged in Styrofoam patty containers, wrapped in cling film and labelled according to formulation and treatment. After the patties were produced, they were placed in plastic containers and transported to the Department of Animal Sciences, Stellenbosch University (SU) for storage at 4 °C (Formulation 1, 2 and 3) and −20 °C (Formulation 4) until analysed.

*Formulation 1: Patty 1 (P1)—Fat% and fat content class/claim*

**Figure 1:**
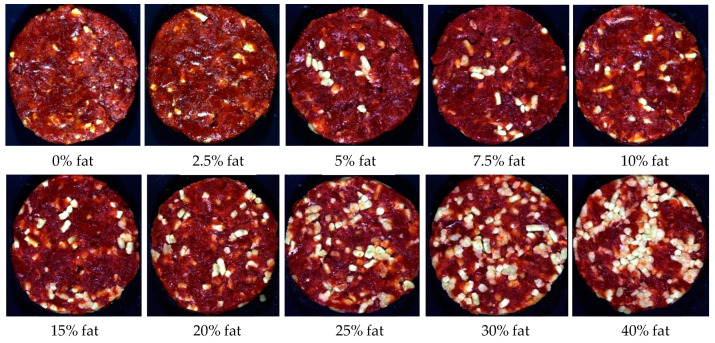
Digital images of the various fat percentages for Patty 1 (ground burger/ground patty).

The patties for the **ground burger category** (Figure 1) were manufactured from beef meat and beef fat only, therefore containing no other added ingredients. These patties had a total meat (TM) content ≥99.6% with varying added fat percentages (treatments) to represent the fat content claim (extra lean, lean, regular) according to regulation [10] (Table S3).

*Formulation 2: Patty 2 (P2)—Species adulteration*

**Figure 2:**
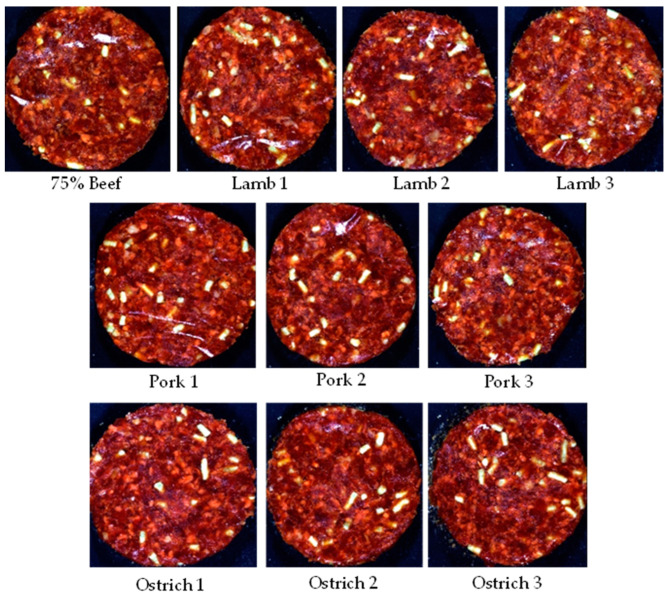
Digital images of the various species substitutions for Patty 2 (burger/patty/hamburger patty/meatball/frikkadel).

The patties for the **burger/patty/hamburger patty category** (Figure 2) were manufactured from beef meat and beef fat (10% added), with a TM content ≥70%. According to the regulation these patties may consist of species mixtures, however a minimum of 75% of the mixture must consist of the meat of the predominant species mentioned on the packaging. Thus, a maximum of 25% thereof may consist of meat from any other animal, bird or game species. These patties may also contain other added ingredients as stated in the regulation (Table S3). For this formulation, the beef fat, spices and water were kept constant across the treatments. These treatments consisted of a control (75% Beef) and nine substitutions with three treatments per species (pork, lamb, ostrich). Treatment 1 and 2 for each species were the ‘authentic’ substitutions, with treatment 3 pertaining to the adulterated patties.

*Formulation 3: Patty 3 (P3)—Textured vegetable protein adulteration*

**Figure 3:**
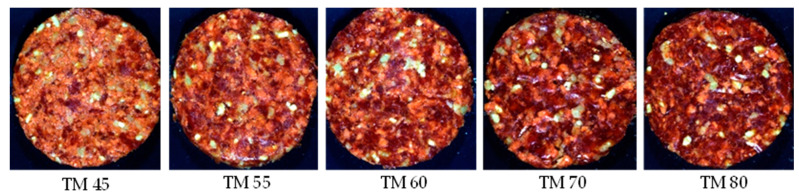
Digital images of the various total meat (TM) percentages for Patty 3 (value burger/value patty/value hamburger/value meatball/value frikkadel/Any other similar name).

The patties for the **value burger/value patty category** (Figure 3) were manufactured from beef meat and beef fat (10% added), with a TM content ≥55% and a minimum total meat equivalent (TME) of 60%. According to the regulation these patties my contain other added ingredients (Table S3). For this formulation, the beef fat and spices were kept constant across the treatments, with the beef meat, water, burger rusk and TVP varying for each treatment. The treatments with a TM% and a TME% above 55% and 60%, respectively, were the authentic patties, with the treatments below these limits pertaining to the adulterated patties.

*Formulation 4: Patty 4 (P4)—Mechanically recovered meat adulteration*

**Figure 4:**
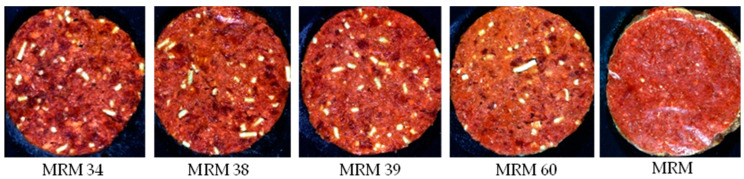
Digital images of the various mechanically recovered meat (MRM) percentages for Patty 4 (economy burger/econo burger/economy patty/econo patty/budget burger/econo hamburger patty/budget hamburger patty/econo meatball/econo frikkadel/Any other similar name).

The patties for the **econo burger/budget burger category** (Figure 4) were manufactured from beef meat, beef fat (10% added) and MRM (added at different percentages), with a TM content ≥35% and a minimum TME of 55%. These patties may also contain other added ingredients as stated in the regulation (Table S3). For this formulation, the beef fat, spices and TVP were kept constant across the treatments, with the beef meat, MRM, water, soya fibre and burger rusk varying for each treatment. The treatments with a TM% and a TME% above 35% and 55%, respectively, were the authentic patties, with the treatments below these limits resulting in the adulterated patties.

### 2.3. Moisture, Fat and Protein Analysis

Patties from each formulation and treatment were tested in duplicate for moisture, fat and protein. The moisture content was determined by drying the homogenized patties at 100 °C for 48 h, according to the Official AOAC Method 934.01 [24]. The crude fat content was determined using the chloroform/methanol (1:2 and 2:1) extraction method as described by Lee et al. [25]. Thereafter, the defatted samples from the chloroform/methanol extraction method were dried, ground to a fine powder and used for the protein content determination. The crude protein was determined using the Dumas combustion method with a LECO FP 528 Nitrogen Analyzer (LECO Corporation, St. Joseph, MI, USA) in accordance with the Official AOAC Method 992.15 [26].

### 2.4. Near-Infrared Hyperspetral Imaging System

Hyperspectral images were acquired with a HySpex SWIR-384 (Norsk Elektro Optikk, Norway) imaging system, in reflectance mode, using the Breeze^®^ (Prediktera) acquisition software. The system’s camera comprised of a mercury–cadmium–telluride (HgCdTe) detector with a maximum frame rate of 400 frames per second (fps). The samples were illuminated with two 150 W halogen lamps, mounted on a laboratory rack 30 cm above the translation stage at a 53° angle. Individual images were acquired using a 30 cm focal length lens at a working distance of 0.208 m and a field of view of 95 mm within the spectral range of 952–2517 nm at increments of 5.45 nm between each of the 288 wavelength channels. These images consisted of 384 pixels in width (x) and varied in length (y). Grey and internal black reference standards were taken every 30 min during the imaging session and used for image correction and calibration. The grey reference standard (Zenith Polymer^®^ Reflectance Standards) comprised of a 50% diffuse reflectance polytetrafluoroethylene (PTFE) surface and the black reference was recorded with the shutter closed. Using the 50% (grey) instead of a 99% (white) reflectance target results in no significant differences in the calculated reflectance values of the sample. The 50% target enables the use of longer integration times, which would normally saturate the 99% target [27]. Because of the longer integration times (3,200 μs), the samples in this study have an increased signal-to-noise ratio.

### 2.5. Image Acquisition and Correction

Prior to imaging, the patties were removed from the 4 °C (Formulation 1, 2 and 3) and −20 °C (Formulation 4) storage and placed at ambient temperature (*ca.* 23 °C) for 30 min. Thereafter, the surface of the patties was blotted dry, with an absorbent tissue paper, to remove the excess moisture before collecting the images. Each patty was placed on a silicone black tray and its entire surface was imaged. This ensured that most of the variation within one sample was recorded. Unique images were obtained for each patty formulation (category), resulting in 200 images per patty category. Throughout this study, the patties were prepared and imaged under the same controlled conditions.

The raw spectral images (*R*_0_) were corrected using a radiometric calibration. The grey and black reference images were used to calculate a relative reflectance image (*R*) using the following equation:

(1) $$R=\frac{R_{0}-D}{W-D}$$

where *R* is the corrected reflectance value, *R*_0_ is the raw irradiance value, *D* (0% reflectance) the dark reference image acquired with the light source off and covering the camera lens with its opaque cap, and *W* (50% reflectance) the white reference image obtained from a grey Teflon calibration tile, subjected to the same lighting conditions as the samples.

The corrected reflectance spectra were then converted to pseudo-absorbance by taking the logarithm of the reflectance values (log 1/*R*). Radiometric calibration from irradiance to radiance to pseudo-absorbance were carried out using the HySpex Ground software v 4.1 (HySpex, Norsk Elektro Optikk, Norway).

### 2.6. Hyperspectral Image Analysis

#### 2.6.1. Analysis Software

The images were analysed using Python v 3.6.5 (G. van Rossum, Python tutorial, Technical Report CS-R9526, Centrum voor Wiskunde en Informatica (CWI), Amsterdam, May 1995) and the PLS_Toolbox [Solo] (Eigenvector Research Inc., Wenatchee, WA) software package.

#### 2.6.2. Image Segmentation (Cleaning) and Extraction of Spectral Data (Mean Spectrum Calculation)

The process of extracting important objects from an input image is referred to as image segmentation. This is extremely useful for identifying regions-of-interest (ROIs) in tested objects in the form of masks and extracting their spectral features.

**Figure 5:**
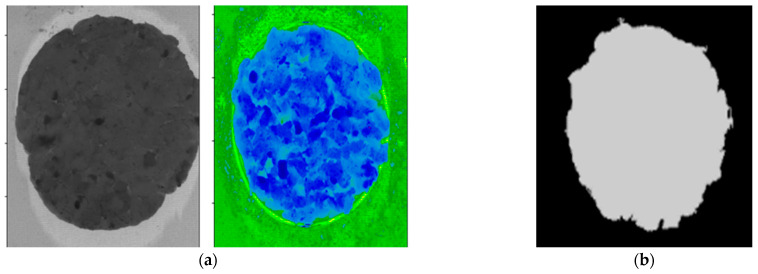
(**a**) A hyperspectral (greyscale and spectral) image of a pure beef burger patty; (**b**) A binary mask image created using thresholding at 952 nm with a value of 1.1.

The goal of segmenting the images was to separate the patties from the background and unwanted pixels. A threshold value was determined based on the pixel intensity values between the patty samples and the image background to isolate the patties from the background. The hyperspectral image was reduced to a binary mask image by thresholding the image at 952 nm (at this wavelength, images provided good contrast between the patty sample and the background) with a value of 1.1. Isolated continuous regions of pixels with similar intensity levels were identified as the objects. In this step, each pixel value was then replaced with either a 0 or 1, where the 0 indicated the background (non-object pixels) and the value 1 the ROI (potential object pixels, i.e., patty), resulting in a binary image or image mask (Figure 5). The background of the original hyperspectral images was removed by multiplying the mask across the hypercube along the λ-dimension. Once only the foreground (ROI) of the images remained, it was used to extract the spectral data from the images. The mean absorbance spectrum for each patty was calculated by averaging the spectra of all pixels within the ROI at each wavelength, in order to perform object-wise analyses.

A spectral matrix was created by combining the extracted spectrum of each sample (patty). In this study, a spectral matrix was created for the identification of the patty categories (800 patty samples as rows and 288 wavebands as columns). Classes for each sample in the matrix was assigned and labelled according to the patty category, e.g., Patty 1, Patty 2, Patty 3 and Patty 4. The mean spectra of each class set (patty category) was computed between 952 and 2517 nm and plotted to investigate, determine and compare the chemical properties.

#### 2.6.3. Pre-Processing

The spectral matrix was subjected to the following pre-processing techniques: (1) mean-centring, (2) standard normal variate (SNV); and (3) SNV + detrending (DT) [28]. These were applied to the spectral data to remove the scattering effects by centring and scaling each individual spectrum (SNV), as well as reduce the baseline shift and curvature (DT). These pre-processing techniques were evaluated in combination with different chemometric- and machine learning algorithms to determine the combination that yield the best results.

#### 2.6.4. Principal Component Analysis

Principal component analysis (PCA) [29] was performed on the mean-centred absorbance spectra. The PCA models were calculated with a maximum of four principal components (PCs) to ensure consistency in the analysis. Subsequently, the PC scores plots and influence plots were used to detect and identify outliers. These outlier samples were removed from the dataset, and the PCA models were recalculated to further explore the data. In addition to outlier detection, the scores and loading line plots were used to visualise clusters of classes and identify important wavelengths, respectively.

#### 2.6.5. Model Development, Calibration and Validation

Prior to model development, the following step was to randomly split the data into a calibration (training) and validation (test) set. Approximately 70% of the original data set was used for training and 30% for testing. The calibration models were calculated with leave-one-out cross-validation (CV), where each sample was left out of the calibration set once and subsequently predicted. Afterwards, the test set samples were predicted to evaluate the performance of the models. The cross-validation and performance measures were also used to evaluate the models as well as identify which pre-treatment/algorithm combination would be most suitable for the present dataset.

Various chemometric- and machine learning algorithms were investigated to develop models to characterise the patty samples and distinguish between the four patty categories (formulations) based on the regulation. The algorithms evaluated were: linear discriminant analysis (LDA) [30]; partial least squares discriminant analysis (PLS-DA) [31]; *K*-nearest neighbour (KNN) [32]; decision trees (D.Trees) [33], random forests (RF) [34] and support vector machines classification (SVM-C) [35].

A grid-search (GridSearchCV) was used to find the best hyperparameters for each model. GridSearchCV performs an exhaustive search across all the classification algorithms’ specified parameter values. The parameters for a specific algorithm varied according to the pre-processing technique used and the categories/classes examined.

#### 2.6.6. Performance Measures

The overall performance of the individual models with the respective pre-processing techniques, were evaluated by performing the following calculations. The classification accuracy (Equation (2)) illustrates the efficacy of the overall model. The false positive error (Equation (3)) describes the misclassification of a negative response (incorrect class) as a positive response (correct class), while the false negative error (Equation (4)) describes the misclassification of a positive response (correct class) as a negative response (incorrect class). The sensitivity (Equation (5)), specificity (Equation (6)) and precision (Equation (7)) were calculated to assess the performance of models for a single class. The sensitivity (true positive rate) and specificity (true negative rate), describes the probability that a correct class and an incorrect class would be correctly classified, respectively, whereas the misclassification rate (classification error) (Equation (8)) describes how often the prediction was incorrect. The precision describes the predictive power of the model by calculating the predicted value for each class.

(2) $$\mathrm{Classification}\;\mathrm{accuracy}\;\left(\%\right)=\frac{\mathrm{TP}+\mathrm{TN}}{\left(\mathrm{TP}+\mathrm{TN}+\mathrm{FP}+\mathrm{FN}\right)}\;\times \;100\%$$

(3) $$\mathrm{False}\;\mathrm{positive}\;\mathrm{error}\;\left(\%\right)=\frac{\mathrm{FP}}{\left(\mathrm{TP}+\mathrm{TN}+\mathrm{FP}+\mathrm{FN}\right)}\;\times \;100\%$$

(4) $$\mathrm{False}\;\mathrm{negative}\;\mathrm{error}\;\left(\%\right)=\frac{\mathrm{FN}}{\left(\mathrm{TP}+\mathrm{TN}+\mathrm{FP}+\mathrm{FN}\right)}\;\times \;100\%$$

(5) $$\mathrm{Sensitivity}\;\mathrm{or}\;\mathrm{Recall}\;\left(\%\right)=\frac{\mathrm{TP}}{\left(\mathrm{TP}+\mathrm{FN}\right)}\;\times \;100\%$$

(6) $$\mathrm{Specificity}\;\left(\%\right)=\frac{\mathrm{TN}}{\left(\mathrm{TN}+\mathrm{FP}\right)}\;\times \;100\%$$

(7) $$\mathrm{Precision}\;\left(\%\right)=\frac{\mathrm{TP}}{\left(\mathrm{TP}+\mathrm{FP}\right)}\;\times \;100\%$$

(8) $$\mathrm{Misclassification}\;\mathrm{rate}\;\left(\%\right)=\frac{\mathrm{FP}+\mathrm{FN}}{\left(\mathrm{TP}+\mathrm{TN}+\mathrm{FP}+\mathrm{FN}\right)}\;\times \;100\%$$

where:

True Positives (TP) = Positive response correctly classified as a positive response

True Negatives (TN) = Negative response correctly classified as a negative response

False Positives (FP) = Negative response incorrectly classified as a positive response

False Negative (FN) = Positive response incorrectly classified as a negative response

#### 2.6.7. Model Limits (Regulation)

A classification accuracy of 75% is considered high in NIR-HSI applications and acceptable for authentication and adulterant identification. However, South African regulations [10] (Table S3) does not allow for any adulterated or mis-labelled raw processed meat products (e.g., raw burger, raw patty, raw hamburger patty) to enter the supply chain and states that: “Anyone who violates or fails to comply with the provisions of these regulations commits an offense and is subject to a fine or imprisonment if convicted” [10]. Therefore, classification accuracies of 100% will be required/mandatory. However, due to this being a preliminary study and the first of its kind in South Africa, the models with accuracies ≥90% were deemed to be acceptable limits for classification of the patty categories.

## 3. Results and Discussion

### 3.1. Moisture, Fat and Protein Analysis

The proximate chemical composition analysis was performed to support the spectral interpretation of the patties, and the results are presented in Table S4. The average values of the proximate composition of the four patty categories were: moisture- [P1 (64.5%); P2 (65%); P3 (64%); P4 (65%)], fat- [P1 (16.8%); P2 (11.9%); P3 (11.6%); P4 (12.4%)] and protein content [P1 (19.5%); P2 (14.4%); P3 (14.2%); P4 (10.4%)], which corresponds to literature. Sheard et al. [36] reported beef burger patties to have a moisture content between 50.4 and 67.3%, whereas the fat content ranged from 7.5 to 29.5%, and the protein content ranged from 13 to 18.2%. The chemical compositional differences observed between the burger patties can be ascribed to the use of different ingredients for the separate formulations and treatments.

### 3.2. Patty Category Determination

This data set consisted of 800 patty samples of four patty categories [Patty 1 (ground burger/patty), Patty 2 (burger patty), Patty 3 (value burger/patty), Patty 4 (econo/budget burger)], with multiple treatments per category (200 patties). The aim of this study was to distinguish between the four patty categories, irrespective of the treatments or the authenticity status.

#### 3.2.1. Spectral Analysis

**Figure 6:**
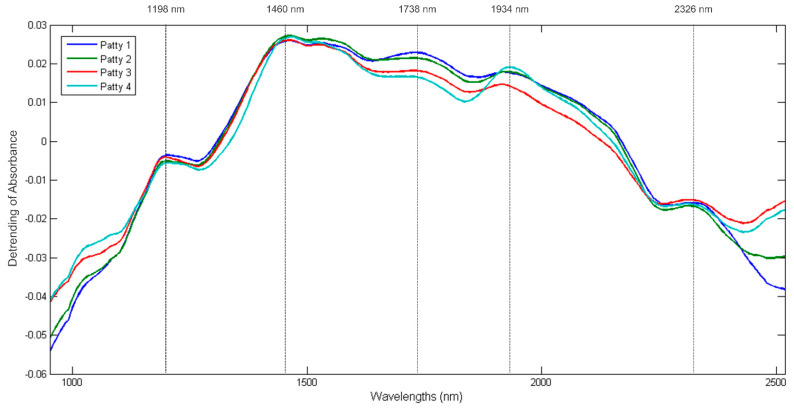
Baseline corrected (detrending) mean pseudo-absorbance spectra for Patty 1 (**blue**), Patty 2 (**green**), Patty 3 (**red**) and Patty 4 (**turquoise**); Five prominent absorption bands were observed at 1198 (fat), 1460 (moisture), 1738 (fat), 1934 (moisture) and 2326 nm (protein).

The mean spectrum of each patty category is shown in Figure 6. The mean spectra of the four patty categories followed a similar trend and exhibited five broad absorption bands at 1198, 1460, 1738, 1934 and 2326 nm. The bands at 1198 nm (C-H stretch 2nd overtone) and 1738 nm (C-H stretch 1st overtone) indicate the presence of fat as specified by Osborne et al. [37] and Murray [38], respectively. The bands observed at 1460 nm (O-H stretch 2nd overtone) [39] and 1934 nm (O-H stretch + O-H deformation) [40] are associated with water and could be related to the moisture content of the samples. Lastly, the band at 2326 nm represents the C-H stretch + C-H deformation related to the CH_2_ group [37] and can be associated with protein as specified by Downey and Beauchêne [41].

The mean spectra of the four patty categories clearly exhibited differences in the absorbance values between all four patty categories at the bands associated with moisture, fat and protein. These differences correlate with the variances observed in the proximate chemical composition analysis results (Table S4) and therefore supports the spectral interpretation of the patties.

#### 3.2.2. Principal Component Analysis

**Figure 7:**
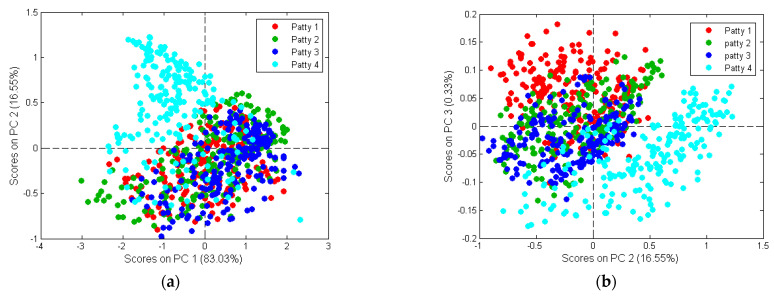
PCA analysis of Patty 1 (**red**), Patty 2 (**green**), Patty 3 (**blue**) and Patty 4 (**turquoise**) categories. Minimal/gradual class separation was observed. Scores illustrated as (**a**) PCA score plot of PC1 (83.03%) vs. PC2 (16.55%); and (**b**) PCA score plot of PC2 (16.55%) vs. PC3 (0.33%).

Figure 7 shows the PCA score plots for the patty categories analysed with the first three PCs. The score plots show clusters with minimal/gradual separation between the different classes. The lack of separation indicates similarity in their spectral signatures; however, there are numerous aspects (e.g., physical and chemical) that could differ, leading to slight spectral differences [42]. These differences are most likely associated with the characteristics and the fluctuations of the macronutrient composition within the patties. Moisture, fat and protein could explain the minimal/gradual separation between the different patty categories.

**Figure 8:**
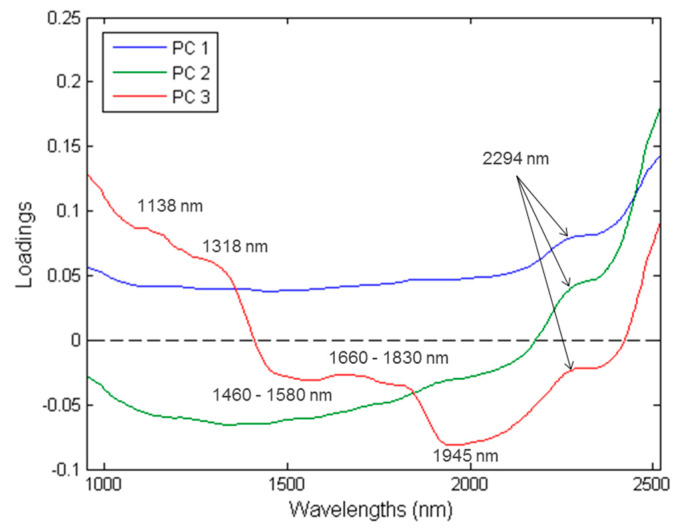
PCA loadings line plot for PC1 (**blue**), PC2 (**green**) and PC3 (**red**) with interpretable bands at 1138, 1318, 1460–1580, 1660–1830, 1945 and 2294 nm.

PC1 explains 83.03% of the total variance (Figure 7), while PC2 and PC3 explains 16.55% and 0.33%, respectively. The loadings (Figure 8) exhibited the variables which are important to a given PC. The highest interpretable loading on PC1 and PC2 were found around 2294 nm (N-H stretch + C=O stretch) related to the amino acids found in protein [37]. PC3 had interpretable loadings at 1138 nm (C-H stretch 2nd overtone), 1318 nm, 1460–1580 nm (N-H stretch 1st overtone), 1660–1830 nm (C-H stretch 1st overtone), 1945 nm (O-H stretch + O-H deformation) and 2294 nm. These bands are associated with fat from beef (1138 nm) [43], protein (1318 nm; 1460–1580 nm) [37,44], fat (1160–1830 nm) [37,39], moisture (1945 nm) [37] and protein (2294 nm) [37]. In other words, Patty 4 (P4) separates from P1, P2, P3 in the direction of PC1 and PC2. Additionally, based on the loadings, this would be due to the difference in protein. P1 and P4 separate in the direction of PC3. By interpreting the loadings and comparing it to the chemical composition analysis results, it was concluded that the separation was due to P1 being higher in fat and protein and P4 higher in moisture. These results agreed with the proximate chemical composition analysis (Table S4) and it is therefore evident from the average moisture-, fat- and protein content of the patties, that these constituents were responsible for the minimal/gradual separation between the classes.

#### 3.2.3. Model Development: Patty Category

LDA, KNN, D.Trees, RF, SVM-C (rbf, linear, poly, sigmoid) and PLS-DA models were calculated to distinguish between the four patty categories (P1–P4). These were done by evaluating the performance of two pre-processing techniques (SNV and SNV+DT) (Table 5).

The SNV and SNV+DT pre-processing techniques gave similar results for the specific chemometric- and machine learning algorithms investigated (Table 5). After investigation, it was concluded that the LDA and SVM-C (rbf, linear, poly) models yielded the best results and could effectively distinguish the four patty categories from one another with accuracies ≥97%. The LDA and SVM-C models all achieved classification accuracies of 100% (calibration), 98–99% (cross-validation) and 97–98% (validation), thus indicating that the models were not over-fitted [45] and effective when predicting the individual patties. These models were then further investigated to determine their performance measures (Table 6, Table 7 and Table 8).

The performance measures exhibit how well the models classified each individual class. The classification of each class was high with the models being capable of distinguishing between the classes with accuracies ranging from 98.3 to 99.2% (Table 6). Due to the models achieving very similar performance measures, the mean performance values for the LDA (Table 7) and each SVM-C (rbf, linear, poly) model (Table 8) was calculated to simplify the interpretation. The mean performance measures for the LDA model (Table 7), shows that it was capable of distinguishing between each patty category with classification accuracies above 98%. The increased sensitivity (100%) and specificity (99.2%) for P4, indicates that the model has a high probability of correctly classifying Patty 4, thus resulting in a classification accuracy of 99.4%. The slightly lower sensitivity for P1 (97.7%), P2 (95.9%) and P3 (95.4%), reveals that the model was to some extent less suited for predicting these three patty classes. This phenomenon can be explained by referring to the calculation of the LDA model. LDA models are constructed using the PC scores, where the algorithm calculates an optimal linear projection between the classes, while keeping the variance within a class to a minimum [30]. Objects (patties) are classified by calculating the distance to the centre of each class. The objects are then assigned to a class to which it has the shortest distance. The scores plot (Figure 7) illustrates an overlap between the samples of Patty 1, Patty 2 and Patty 3, with a slightly larger number of Patty 3 samples displaying a close distance to the samples of Patty 1 and Patty 2. Therefore, Patty 3 resulted in a lower classification accuracy as these patties were assigned to the predominant class, Patty 1 and/or Patty 2. Hence, explaining why the model is slightly less suited for predicting the Patty 3 category samples.

The overall mean classification accuracies for the individual patty classes of the SVM-C models for the rbf (>97.1%), linear (>98.5%) and polynomial (>98.1%) kernel functions, suggested that the model calculated with the linear kernel would provide slightly better classification (Table 8). A kernel is a similarity function which calculates how similar two inputs are and is used for separating hyperplanes, to evaluate the robustness of the classifier model. SVM is a maximal margin classifier where the algorithm aims to separate the different categories/classes in a dataset by placing a *separating line* in the middle of the *margin*. The empty space between the boundaries, known as the *maximum margin* or *optimal margin hyperplane*, indicates the maximum separation between two groups. The data points touching the boundary of the margin are known as the *support vectors* [46], which serve as training patterns and convey all pertinent information regarding the classification problem [47]. In the end, SVM is a distance-based approach which calculates the optimal distance between data points and the hyperplane [46].

The mean performance measures for the SVM-C (linear) model given in Table 8, shows that the P4 class achieved the highest mean classification accuracy (99.8%), followed by P2 (99.0%), P3 (99.0%) and P1 (98.5%). The model also revealed that the classification of each class was nearly perfect, with the sensitivity (>97.0%), specificity (>98.3%) and precision (>95.5%) confirming this observation. The slightly lower classification accuracy of P1, P2 and P3 was ascribed to the increased false positive- and false negative error due to objects being misclassified. The lack of separation can be attributed to the patties’ spectral similarities which corresponds to their similar proximate chemical composition results as reported in Table S4. The sensitivity and specificity of Patty 1 was 99.2% and 98.3%, respectively. This suggests that the model is equally sensitive for predicting a true positive (Patty 1) as correct, as it is specific when predicting a true negative (Patty 2, Patty 3 and Patty 4) as correct. The sensitivity of Patty 2 and Patty 3 is 97.5% and 97.0%, respectively. In addition, the specificity is 99.4% and 99.1%, therefore revealing that the model is less sensitive and more specific when classifying these patties. The results for the SVM-C models given in Table 8, shows that this machine learning algorithm was capable of distinguishing between each patty category.

From both the LDA and the SVM-C (rbf, linear, poly) results it was evident that these models were capable of accurately distinguishing Patty 4 from the other three patties, with a misclassification rate below 0.6%. The PCA scores (Figure 7) and loadings (Figure 8) support these results, as a slight separation was observed between Patty 4 and the other three patties. The separation and correct classification of P4 was mainly attributed to the lower protein content, which accounted for the spectral differences. The protein content of Patty 4 (10.7%) was considerably different, compared to the other patties [Patty 1 (19.5%), Patty 2 (14.4%), Patty 3 (14.2%)], and a correlation was observed in the chemical results (Table S4). Therefore, the results illustrate that the above-mentioned models were able to predict the different patty classes, with relatively high accuracies, which deemed the models to be effective.

Although the D.Trees- and RF models both exhibited calibration accuracies of 100%, indicative of an effective model, the cross-validation [82–83% (D.Trees); 88–90% (RF)] and validation accuracies [74–76% (D.Trees); 83–93% (RF)] decreased (Table 5). This indicated that the models were over-fitted [45] and therefore not effective. Over-fitting is a known drawback of decision trees, especially when dealing with many features. This dataset consisted of 288 features (wavebands), therefore explaining the tendency to overfit the models. A decision tree is a flowchart-like tree structure where the basic development involves the splitting of the predictor space, using recursive binary splitting, into a number of simple regions for all the possible outcomes [48]. The binary splits are made using the classification error rate, which are the number of training observations in that given region not belonging to the most common class. An unknown observation in a given region is assigned to the most common class in that region. Although decision trees are simple and useful for interpretation, they lack in predictive accuracy compared to other supervised learning approaches [48]. Hence, the prediction accuracy can substantially be improved by producing multiple trees and aggregating them using a method like random forests. This phenomenon was supported by the improved cross-validation and validation results observed in Table 5 for the RF models. Although, random forests is a collective of decision trees, how the trees are constructed differ. For decision trees, each tree-node is selected using all the features to gain the maximum amount of information. While random forests only consider a small subset of features when constructing a node, thus resulting in some loss of interpretation [48]. This phenomenon could explain the improved classification performances of the RF models.

The KNN, SVM-C (sigmoid) and PLS-DA models exhibited unsatisfactory classification and discrimination results (Table 5). This can be explained by referring to how the individual models are computed. KNN is a distance-based, non-parametric discriminant classification method [49], performed on the PC scores. The distance between an unknown (test set) and a set of samples with a known class membership (training set) is calculated to classify the unknown. The closest K samples, determined by GridSearchCV, and majority voting were used to make a classification. Therefore, the classification of the individual patties was impaired as a result of the close distance and overlap between the four classes due to the minimal separation observed in the scores plot (Figure 7).

The SVM-C (sigmoid) model’s decreased accuracy could be explained by the fact that the sigmoid kernels are less adaptable than rbf kernels, resulting in higher bias when computing the separating hyperplanes [46]. Optimal model performances can be achieved when the parameters *penalty coefficient C* and *gamma* are optimised using a grid-search procedure. The *C* value is an important parameter as it determines the size of the margins of the hyperplane and to what extent the model under-fits or over-fits the data. A large *C* will result in broad ‘soft margins’, with a high tolerance for observations violating the constraints. Consequently, the ‘soft margin’ allows for the misclassification of some training samples, thus resulting in a better overall model fit [50]. As *C* decreases, the tolerance for observations violating the constraints decreases, and the margins narrows, resulting in ‘hard margins’ and over-fit data. Both SVM-C (sigmoid) models had low *C* values, which could explain the reduced classification model performances.

The four-way PLS-DA model was constructed, using binary dummy variables, to indicate the presence or absence of a specific class during the modelling process. For example, a value of one was assigned if the spectrum belonged to the correct group, and a value of zero if it did not [42]. While achieving separations of two classes is known to be relatively easy [27], the results (Table 5) in the current study support the notion that the separation of multiple classes is challenging. Although many studies have utilised a single globally optimised model to discriminate between two, three or even four classes [27], the development of such models is not always straight forward. This type of multi-class model approach is only possible once all the classes are fully separable using the chosen set of spectral features. This requirement is frequently not met, particularly when working with heterogeneous samples and closely related classes [27], as was observed in the scores plot (Figure 7). For this reason, the PLS-DA models exhibited unsatisfactory discrimination accuracies.

## 4. Conclusions

The South African meat industry currently relies on destructive and time-consuming techniques which require complex laboratory procedures to authenticate processed meat products. NIR hyperspectral imaging has been considered as an automated alternative to improve and modernise this process. Although the initial investment cost for a HIS-system is substantial (prices start at $28k USD) [51], the economic importance of meat and processed meat products as well as the wide-ranging benefits of improved authentication procedures could make up for this costly price tag. In addition, there will be savings on reagents, increased speed of analysis and overall higher throughput. A series of models, using various chemometric- and machine learning algorithms, were calculated in order to classify each object as either of the four patty categories. The SVM-C (linear) models distinguished the four patty categories with classification accuracies ≥98.5%.

This study sets the benchmark, as it is the first time that NIR-HSI was investigated for the rapid detection, classification and categorisation of beef patties based on the South African regulations and different ratios of water:protein:fat. NIR-HSI is a suitable eco-friendly technique and shows potential to rapidly and accurately distinguish between categories of processed beef burger patties consisting of various ingredients, species and adulterants. Additionally, NIR-HSI has excellent application prospects since this approach adheres to the fundamental principles of ‘green science’, reducing waste formation and not requiring the use of chemical reagents or solvents. Furthermore, this study has the potential of providing an alternative technique to the current AOAC-recommended conventional, manual, destructive and time-consuming methods, thus contributing to the authenticity and fair-trade of processed meat products locally and internationally. However, the South African legislation is strict, and the classification of the patty categories must be refined further before the meat sector considers spectral imaging. Therefore, the next step for this research would be to investigate the authentication and adulterant detection/quantification of the patties within each individual patty category.

## Figures and Tables

**Table 1:** Formulation of treatments for Patty 1 (ground burger/ground patty).

Ingredients	Treatments (Added Fat %)									
	0% Fat	2.5% Fat	5% Fat	7.5% Fat	10% Fat	15% Fat	20% Fat	25% Fat	30% Fat	40% Fat
Lean Beef (%)	100	97.5	95	92.5	90	85	80	75	70	60
Beef Fat (%)	0	2.5	5	7.5	10	15	20	25	30	40
Total (g)	100	100	100	100	100	100	100	100	100	100

**Table 2:** Formulation of treatments for Patty 2 (burger/patty/hamburger patty).

Ingredients	Treatments (Added Species %)									
	75% Beef (Control)	18.75% Pork 1	7.5% Pork 2	33.75% Pork 3	18.75% Lamb 1	7.5% Lamb 2	33.75% Lamb 3	18.75% Ostrich 1	7.5% Ostrich 2	33.75% Ostrich 3
Lean Beef (%)	65	46.25	57.5	31.25	46.25	57.5	31.25	46.25	57.5	31.25
Beef Fat (%)	10	10	10	10	10	10	10	10	10	10
Spice Pack (%)	10	10	10	10	10	10	10	10	10	10
Water (%)	15	15	15	15	15	15	15	15	15	15
Lean Pork (%)	0	18.75	7.5	33.75	0	0	0	0	0	0
Lean Lamb (%)	0	0	0	0	18.75	7.5	33.75	0	0	0
Lean Ostrich (%)	0	0	0	0	0	0	0	18.75	7.5	33.75
Total (g)	100	100	100	100	100	100	100	100	100	100

**Table 3:** Formulation of treatments for Patty 3 (value burger/value patty).

Ingredients	Treatments (Added Species %)				
	TM 45	TM 55	TM 60	TM 70	TM 80
Lean Beef (%)	35	45	50	60	70
Beef Fat (%)	10	10	10	10	10
Spice Pack (%)	10	10	10	10	10
Water (%)	35.5	30	25.5	13.75	9
Burger Rusk (%)	5	0	0	0	0
TVP (%)	4.5	5	4.5	6.25	1
Total (g)	100	100	100	100	100
TM%	45	55	60	70	80
TME%	55.8	67	70	85	82.4
Authenticity	Adulterated	Authentic	Authentic	Authentic	Authentic

**Table 4:** Formulation of treatments for Patty 4 (econo burger/budget burger).

Ingredients	Treatments (Added Species %)				
	TM 45	TM 55	TM 60	TM 70	TM 80
MRM (%)	50	30.2	15.8	21	21
Lean Beef (%)	0	10	15	25	30
Beef Fat (%)	0	10	10	10	10
Spice Pack (%)	10	10	10	10	10
Water (%)	35	33.8	41.2	29	24
Soya Fibre (%)	0	1	1	0	0
Burger Rusk (%)	0	0	2	0	0
TVP (%)	5	5	5	5	5
Total (g)	100	100	100	100	100
TM%	0	20	25	35	40
TME%	55	55	45	63	67.3
Authenticity	Adulterated	Adulterated	Adulterated	Authentic	Authentic

**Table 5:** An overview of the accuracies for the developed models, pre-processed with SNV and SNV + detrend, to distinguish between the patty categories. The best predictions are indicated in bold.

Pre-Processing	Model	Classification Accuracy (%)								
		LDA (2 PCs)	KNN (*k* = 5)	D.Trees	RF (N^o^ = 100)	SVM-C (rbf) C: 1000 Ƴ: 0.001	SVM-C (Linear) C: 10 Ƴ: 1	SVM-C (Poly) C: 0.1 Ƴ: 0.1	SVM-C (Sigmoid) C: 10 Ƴ: 0.001	PLS-DA (7 LVs)
SNV	CAL	**100**	87	100	100	**100**	**100**	**100**	65	87
	CV	**98**	77	83	88	**98**	**99**	**99**	65	86
	VAL	**97**	76	74	83	**97**	**98**	**97**	57	91
		LDA (2 PCs)	KNN (*k* = 3)	D.Trees	RF (N^o^ = 100)	SVM-C (rbf) C: 1000 Ƴ: 0.001	SVM-C (linear) C: 100 Ƴ: 1	SVM-C (poly) C: 0.1 Ƴ: 0.1	SVM-C (sigmoid) C: 100 Ƴ: 0.001	PLS-DA (5 LVs)
SNV+DT	CAL	**100**	94	100	100	**100**	**100**	**100**	69	76
	CV	**98**	86	82	90	**98**	**99**	**99**	64	56
	VAL	**97**	83	76	93	**97**	**98**	**97**	62	81

(LDA) Linear discriminant analysis; (KNN) *K*-nearest neighbours; (D.Trees) Decision trees; (RF) Random forests; (SVM-C) Support vector machines calibration; (PLS-DA) Partial least squares discriminant analysis; (SNV) Standard normal variate; (DT) Detrend; (CAL) Calibration; (CV) Cross-validation; (VAL) Validation; (PCs) Principal components; (LVs) Latent variables; (N^o^) Number of trees.

**Table 6:** The overall performance measures used to assess the validation results for the classification of patties into four classes.

Pre-Processing	Classification Accuracy (%)	FP Error (%)	FN Error (%)	Sensitivity (%)	Specificity (%)	Precision (%)	Misclassification (%)
Linear Discriminant Analysis							
SNV	98.7	0.6	0.6	97.6	99.2	97.5	1.3
SNV+DT	98.3	0.8	0.9	96.8	98.9	96.7	1.7
Support Vector Machines Classification (rbf)							
SNV	98.3	0.9	0.8	96.7	98.9	96.9	1.7
SNV+DT	98.7	0.6	0.6	97.6	99.1	97.6	1.3
Support Vector Machines Classification (linear)							
SNV	99.2	0.4	0.3	98.3	99.4	98.5	0.8
SNV+DT	99.0	0.5	0.5	98.0	99.3	98.1	1.0
Support Vector Machines Classification (polynomial)							
SNV	98.6	0.8	0.8	97.1	99.0	97.4	1.4
SNV+DT	98.5	0.7	0.7	97.2	99.0	97.1	1.5

(SNV) Standard normal variate; (DT) Detrend; (FP) False positive; (FN) False negative.

**Table 7:** The overall mean performance measures of the combined SNV and SNV+DT corrected data for the LDA models used to assess the validation results for the classification of patties into four classes.

Class	Classification Accuracy (%)	FP Error (%)	FN Error (%)	Sensitivity (%)	Specificity (%)	Precision (%)	Misclassification (%)
P1	98.3	1.1	0.6	97.7	98.6	96.2	1.7
P2	98.3	0.6	1.1	95.9	99.2	97.5	1.7
P3	98.1	0.6	1.3	95.4	99.1	97.7	1.9
P4	99.4	0.6	0	100	99.2	97.2	0.6

(P1) Patty 1; (P2) Patty 2; (P3) Patty 3; (P4) Patty 4; (FP) False positive; (FN) False negative.

**Table 8:** The overall mean performance measures of the combined SNV and SNV+DT corrected data for the SVM-C models used to assess the validation results for the classification of patties into four classes.

Class	Classification Accuracy (%)	FP Error (%)	FN Error (%)	Sensitivity (%)	Specificity (%)	Precision (%)	Misclassification (%)
Support Vector Machines Classification (rbf)							
P1	98.6	1.1	0.4	98.4	98.5	96.2	1.4
P2	97.1	1.1	1.9	92.5	98.6	95.7	2.9
P3	98.5	0.9	0.6	97.9	98.8	97.0	1.5
P4	100	0	0	100	100	100	0
Support Vector Machines Classification (linear)							
P1	98.5	1.3	0.2	99.2	98.3	95.5	1.5
P2	99.0	0.4	0.6	97.5	99.4	98.3	1.0
P3	99.0	0.2	0.9	97.0	99.7	99.2	1.0
P4	99.8	0	0	99.0	100	100	0.2
Support Vector Machines Classification (polynomial)							
P1	98.1	1.1	0.9	96.9	98.6	96.1	1.9
P2	98.4	0.4	1.3	95.0	99.5	98.3	1.6
P3	98.1	1.3	0.6	97.7	98.3	95.5	1.9
P4	99.6	0.2	0.2	99.0	99.8	99.0	0.4

(P1) Patty 1; (P2) Patty 2; (P3) Patty 3; (P4) Patty 4; (FP) False positive; (FN) False negative.

## Data Availability

Not applicable.

## Supplementary Materials

The following supporting information can be downloaded at: https://www.mdpi.com/article/10.3390/s23020697/s1, Figure S1: A schematic of the experimental design, formulation and treatments for Patty 1 (ground burger/ground patty); Figure S2: A schematic of the experimental design, formulation and treatments for Patty 2 (burger/patty/hamburger patty/meatball/frikkadel); Figure S3: A schematic of the experimental design, formulation and treatments for Patty 3 (value burger/value patty/value hamburger/value meatball/value frikkadel/Any other similar name); Figure S4: A schematic of the experimental design, formulation and treatments for Patty 4 (economy burger/econo burger/economy patty/econo patty/budget burger/econo hamburger patty/budget hamburger patty/econo meatball/econo frikkadel/Any other similar name); Table S1: Minimum size of a primary sample; Table S2: Recommended methods of analysis; Table S3: Regulations regarding the classification and compositional specifications of raw beef patties; Table S4: An overview of the proximate chemical composition analysis results (means ± SD) for the moisture-, fat- and protein content (%) of the various patties.
