# Supplementary material for: Raw Beef Patty Analysis Using Near-Infrared Hyperspectral Imaging: Identification of Four Patty Categories
Source: Sensors (Basel). 2023 Jan 7;23(2):697. doi: 10.3390/s23020697 (PMC9867321; doi:10.3390/s23020697)
Supplement: Supplementary file 1 [file sensors-23-00697-s001.zip › sensors-2034474-supplementary.pdf]

Supplementary Materials

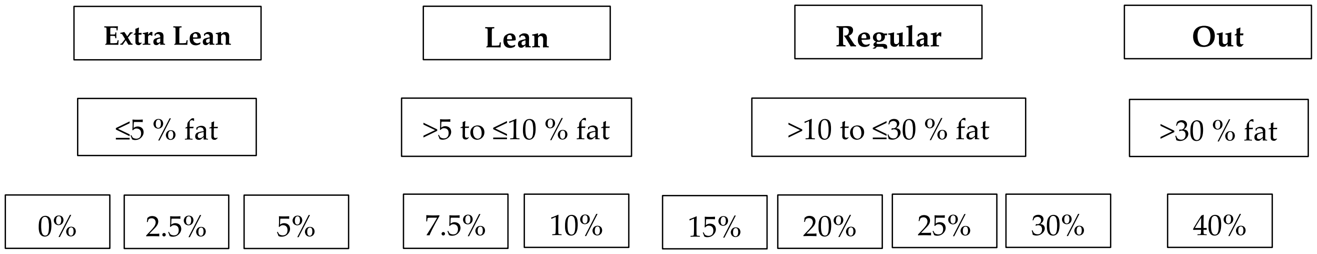

Figure S1: A schematic of the experimental design, formulation and treatments for Patty 1 (ground burger/ground patty).

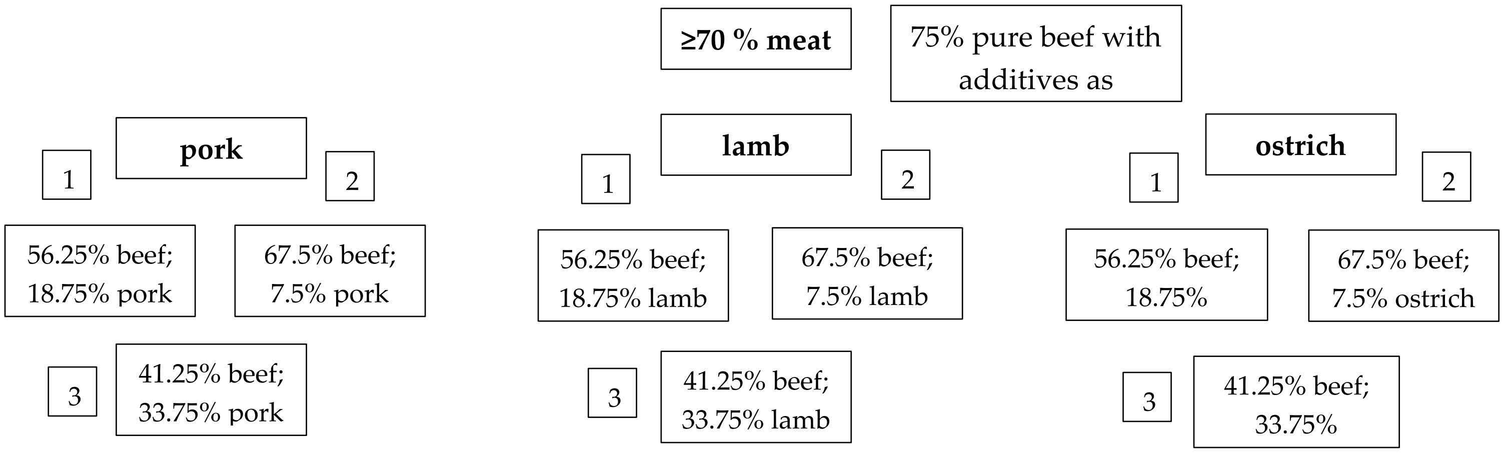

Figure S2: A schematic of the experimental design, formulation and treatments for Patty 2 (burger/patty/hamburger patty/meatball/frikkadel).

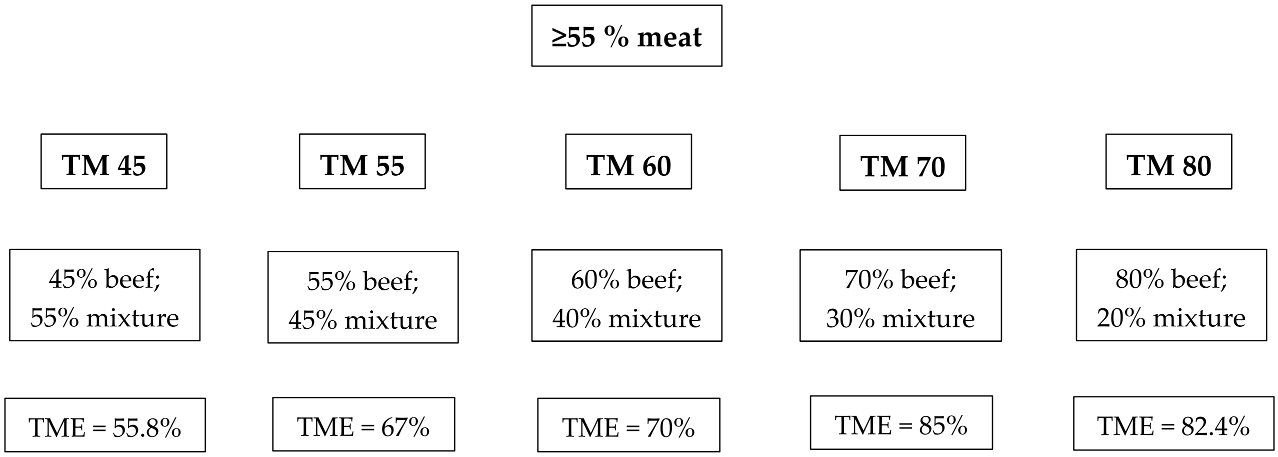

Figure S3: A schematic of the experimental design, formulation and treatments for Patty 3 (value burger/value patty/value hamburger/value meatball/value frikkadel/Any other similar name).

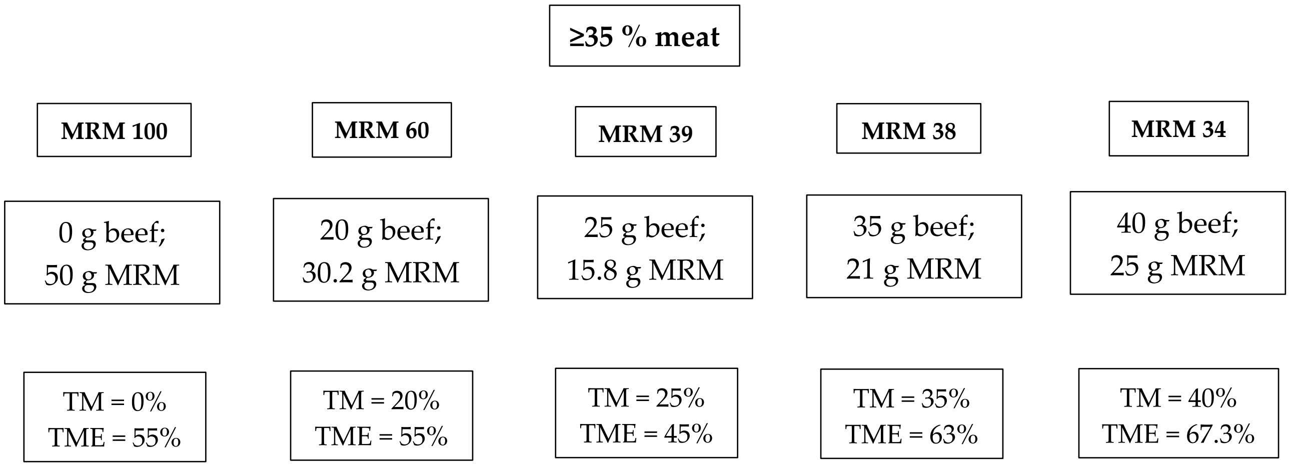

Figure S4: A schematic of the experimental design, formulation and treatments for Patty 4 (economy burger/econo burger/economy patty/econo patty/budget burger/econo hamburger patty/budget hamburger patty/econo meatball/econo frikkadel/Any other similar name).

Obtaining a primary sample

14. (1) (a) Samples drawn for the purpose of analysis shall, depending on the capacity of the container, consist of the corresponding number of containers set out in column 2 of the table below:

Table S1 Minimum size of a primary sample (DALRRD, 2022).

Capacity of the container	Minimum size of primary sample
(i) ≤ 250 g	(i) The minimum number of containers that will result in a combined total mass of at least 250 g; or
(ii) > 250 g ≤ 2 kg	(ii) The minimum number of containers that will result in a combined total mass of at least 1 kg; or
(iii)> 2 kg	(iii) One container

(b) The number of containers so obtained shall constitute a primary sample.

(c) Each container in the primary sample shall be placed in a plastic bag (or any other suitable outer container) that has been properly closed and sealed to ensure the product is tamper-proof.

(d) Each sample shall be clearly and adequately labelled to ensure traceability and correct identification.

(e) An inspector may increase the number of containers that constitute a primary sample to also provide additional samples to perform e.g. counter analysis by a different (second) laboratory, etc.

Methods of analysis

15. (1) The test samples shall be prepared in accordance with the latest version of the Association of Analytical Communities' (AOAC) methods on the preparation of test samples for meat and meat products, i.e. AOAC 983.18, or any other international recognised alternative method.

(2) The determination of the composition of the raw processed meat products shall be done in accordance with the methods set out in table below, or any other international recognised alternative methods providing equivalent results: Provided that at all times the most recently published version of the listed methods or their alternatives shall be used:

Table S2 Recommended methods of analysis (DALRRD, 2022).

Parameter	Test method	Principle	Type
Meat content	AOAC 928.08	Nitrogen determination, calculation	I
	ISO 937:1978 (Protein [conversion factor 6.25]	Titrimetry, Kjeldahl digestion	II
Fat content	ISO 1443-1973 (Codex general method for processed meat and poultry products)	Gravimetry (Extraction)	I
	AOAC 960.39	Soxhlet Ether Extraction	-
Moisture content	ISO 1442:1997	Moisture determination	-
	AOAC 985.14	Moisture in Meat and Meat Product	-
Soya protein content	AOAC 988.10	Protein/Soy Protein,<br>Enzyme-Linked Immunosorbent Assay	-
Starch content	ISO 13965-1998	Determination of Starch and Glucose content – Enzyme method	-
Calcium content, Determination of calcium content for mechanically recovered meat (MRM) only	PEARSON – Pearson's Chemical Analysis of Foods (8th edition), 1981 H. Egan, R.S. Kirk and R. Sawyer – Longman Scientific. Pages 27-28	Determination of calcium by atomic absorption spectrophotometry	-
	AOAC 983.19	Determination of calcium in Mechanically Separated Poultry and Beef	-

Table S3: Regulations regarding the classification and compositional specifications of raw beef patties (DALRRD, 2022).

CATEGORY/PRODUCT NAME	PERMISSIBLE INGREDIENTS	FAT CONTENT CLAIM	FAT CONTENT (%) (as analysed)	TOTAL MEAT CONTENT (%)
1	2	3	4	5
1. Ground Burger / Ground Patty	a) Shall be manufactured from meat only<br>b) Shall contain no edible or inedible offal, or any other added ingredients	Extra Lean, Extra Trim, Extra trimmed of fat or any similar wording	≤ 5	≥ 99.6
		Lean, Trim, Trimmed of fat or any similar wording	> 5 to ≤ 10	
		Regular	> 10 to ≤ 30	
2. Burger/Patty/ Hamburger Patty/ Meatball/Frikkadel	a) Shall contain no added ingredients other than –<br>i. cereal and/or starch;<br>ii. vinegar, spices, herbs and/or salt;<br>iii. food additives; and<br>iv. water	Extra Lean, Extra Trim, Extra trimmed of fat or any similar wording	≤ 5	≥ 70
	b) Shall contain no edible or inedible offal	Lean, Trim, Trimmed of fat or any similar wording	> 5 to ≤ 10	
	c) Shall contain no mechanically recovered meat	Regular	> 10 to ≤ 30	
	d) Shall contain no colourants<br>e) Shall contain no vegetable protein<br>f) May contain other foodstuffs			
3. Value Burger/ Value Patty/ Value Hamburger/ Value Meatball/ Value Frikkadel/ Any other similar name	a) Shall contain no added ingredients other than –<br>i. cereal or starch and/or vegetable protein;<br>ii. vinegar, spices, herbs and/or salt;<br>iii. food additives; and<br>iv. water<br>b) Shall have a minimum total meat equivalent of 60 percent<br>c) Shall contain no inedible offal<br>d) May contain –<br>i. mechanically recovered meat;<br>ii. edible offal;<br>iii. colourants; and<br>iv. other foodstuffs	*	≤ 30	≥ 55

Table S3: Continued.

CATEGORY/PRODUCT NAME	PERMISSIBLE INGREDIENTS	FAT CONTENT CLAIM	FAT CONTENT (%) (as analysed)	TOTAL MEAT CONTENT (%)
1	2	3	4	5
4. Economy Burger/ Econo Burger/ Economy Patty/ Econo Patty/ Budget Burger/ Econo Hamburger patty/Budget Hamburger patty/ Econo Meatball/ Econo Frikkadel/ Any other similar name	a) Shall contain no added ingredients other than – i. cereal or starch and/or vegetable protein; ii. vinegar, spices, herbs and/or salt; iii. food additives; and iv. water<br>b) Shall have a minimum total meat equivalent of 55 percent<br>c) Shall contain no inedible offal<br>d) May contain – i. mechanically recovered meat; ii. edible offal; iii. colourants; and iv. other foodstuffs	*	≤ 30	≥ 35

* No specification

Table S4: An overview of the proximate chemical composition analysis results (means ± SD) for the moisture-, fat- and protein content (%) of the various patties.

	Moisture (%)	Crude Fat (%) (Acid Hydrolysis)	Crude Protein (%)
	Patty 1		
P1 0%	75.06 ± 0.32	2.03 ± 0.67	23.21 ± 0.15
P1 2.5%	72.96 ± 0.43	2.78 ± 1.87	22.43 ± 0.32
P1 5%	71.62 ± 0.84	5.5 ± 1.66	21.85 ± 0.25
P1 7.5%	70.29 ± 0.04	7.64 ± 1.86	21.28 ± 0.36
P1 10%	66.88 ± 0.74	12.3 ± 0.28	20.7 ± 0.89
P1 15%	64.66 ± 1.15	17.25 ± 0.21	19.55 ± 1.89
P1 20%	63.3 ± 0.03	20.9 ± 1.06	18.4 ± 1.92
P1 25%	57.48 ± 1.78	25.5 ± 1.24	17.25 ± 1.86
P1 30%	54.76 ± 0.43	32.1 ± 1.46	16.1 ± 1.57
P1 40%	47.86 ± 0.24	41.8 ± 2.61	13.8 ± 0.78
**AVERAGE**	**64.49**	**16.78**	**19.45**
	Patty 2		
P2 Beef	65.37 ± 0.7	11.95 ± 0.84	14.95 ± 0.6
P2 Pork 1	64.84 ± 0.29	11.86 ± 0.76	13.83 ± 0.35
P2 Pork 2	64.87 ± 0.38	11.91 ± 1.03	14.5 ± 0.5
P2 Pork 3	64.54 ± 0.51	11.78 ± 1.37	12.93 ± 0.09
P2 Lamb 1	62.76 ± 1.53	12.33 ± 1.45	14.39 ± 0.72
P2 Lamb 2	64.59 ± 0.28	12.1 ± 1.87	14.73 ± 0.17
P2 Lamb 3	62.32 ± 0.29	12.63 ± 1.05	13.94 ± 0.02
P2 Ostrich 1	66.55 ± 0.05	11.69 ± 2.88	14.76 ± 0.61
P2 Ostrich 2	66.43 ± 0.18	11.85 ± 2.9	14.88 ± 0.16
P2 Ostrich 3	67.6 ± 0.45	11.48 ± 4.52	14.61 ± 0.22
**AVERAGE**	**64.99**	**11.96**	**14.35**

Table S4: Continued.

	Patty 3		
P3 TM 45	62.84 ± 0.76	11.1 ± 0.47	10.37 ± 0.02
P3 TM 55	66.88 ± 0.41	11.41 ± 2.19	12.93 ± 0.52
P3 TM 60	65 ± 0.41	11.55 ± 0.53	13.82 ± 0.89
P3 TM 70	61.6 ± 0.13	11.88 ± 0.12	17.02± 1.05
P3 TM 80	63.65 ± 0.03	12.11 ± 0.43	16.62 ± 1.15
**AVERAGE**	**64.00**	**11.61**	**14.15**
	Patty 4		
P4 MRM 100	72.24 ± 0.1	7.06 ± 1.51	10.08 ± 0.3
P4 MRM 60	64.53 ± 3.23	14.59 ± 4.65	9.41 ± 1.85
P4 MRM 39	64.30 ± 1.03	12.72 ± 2.39	8.4 ± 2.22
P4 MRM 38	63.26 ± 1.16	13.75 ± 2.62	11.48 ± 1.55
P4 MRM 34	60.68 ± 0.31	13.9 ± 4.82	12.63 ± 1.79
**AVERAGE**	**65.00**	**12.40**	**10.40**
